# Charging Ex Doctors

**Published:** 1889-02

**Authors:** 


### EDITORIALS AND MISCELLANEOUS.

CHARGING EX DOCTORS, ETC.—The question is again submitted to us, Are ex doctors, or those who have retired from the practice entitled to the courtesy usually allowed to medical men of exemption from charge for medical services? We annswer that the ex doctor is not entitled to free medical services, either for himself or family. Such courtesy in any and all cases is based upon the idea of reciprocity, and does not hold outside of the immediate community where the party resides, although it has become a custom to extend it to cases where a visiting brother or a sojourner happens to be taken ill when away from home. In all such cases the principle of reciprocity exists in the fact that those who thus serve him may themselves at some time be similarly situated when away from home, and may need the courtesies due from the brotherhood. Yet it is becoming, and proper always to manifest our gratitude by a suitable present or token to the brother who has served us in sickness—especially is this incumbent upon us when there is no probability that we can ever reciprocate the favor.

The question as to serving ministers gratuitously is also frequently raised. The rule here is different in different communities. There is no obligation resting upon the physician more than any other class of men to render gratuitous services to ministers. If the minister gets a living salary, and a majority of them are as well to do as to worldly support as other people, then why should he, more than other people, be entitled to the time and labor of the physician without compensating him. There is no good reason why the doctor more than the merchant or the grocer should extend gratuities to the minister, nor should the minister expect it. The truth is that too many people expect gratuitous services from medical men.

There are cases where Ministers like other classes of men may need our sympathy and aid, and the physician is usually in a position to judge of the worthiness of such cases: and if he sees proper in any case to practice for his preacher without charge it is his pleasure and privilege to do so; but what we claim is that the preacher has uo more right to expect gratuitous service than other people.

There is no occupation in life so much imposed on as that of the physician A large percentage of his time and labor is necessarily devoted to the poor, to whose demands both the dictates of humanity and public sentiment constrain him to yield. This constitutes a severe draught upon his time, his anxieties, his labors and his health. And yet like other citizens he pays his taxes, his preacher, and performs all the duties of good citizenship. It would seem then, that there is no reason or justice in demanding further gratuities at his hands.

